# Further Evidence of Benefits to Mood and Working Memory from Lipidated Curcumin in Healthy Older People: A 12-Week, Double-Blind, Placebo-Controlled, Partial Replication Study

**DOI:** 10.3390/nu12061678

**Published:** 2020-06-04

**Authors:** Katherine H. M. Cox, David J. White, Andrew Pipingas, Kaylass Poorun, Andrew Scholey

**Affiliations:** Centre for Human Psychopharmacology, Swinburne University, Hawthorn, Victoria 3122, Australia; kcox@swin.edu.au (K.H.M.C.); dawhite@swin.edu.au (D.J.W.); apipingas@swin.edu.au (A.P.); kpoorun@swin.edu.au (K.P.)

**Keywords:** curcumin, working memory, mood, cognition

## Abstract

Curcumin (a flavonoid isolated from turmeric) affects several processes involved in neurocognitive aging. We have previously reported that short term (4-weeks) administration of a highly bioavailable curcumin preparation (Longvida©) improved working memory and reduced fatigue and stress reactivity in a healthy older cohort. The present trial (ACTRN12616000484448) was a partial replication study, evaluating similar effects at 4 and 12-weeks Longvida© supplementation. A double-blind, placebo-controlled, parallel-groups trial was conducted. Eighty participants aged 50–80 years (mean = 68.1, SD = 6.34) were randomised to receive Longvida© (400 mg daily containing 80 mg curcumin) or a matching placebo. Assessment took place at baseline then following 4 and 12 weeks treatment. Outcome measures included cognitive performance, mood and biomarkers. Compared with placebo, curcumin was associated with several significant effects. These included better working memory performance at 12-weeks (Serial Threes, Serial Sevens and performance on a virtual Morris Water Maze), and lower fatigue scores on the Profile of Mood States (POMS) at both 4 and 12-weeks, and of tension, anger, confusion and total mood disturbance at 4-weeks only. The curcumin group had significantly elevated blood glucose. These results confirm that Longvida© improves aspects of mood and working memory in a healthy older cohort. The pattern of results is consistent with improvements in hippocampal function and may hold promise for alleviating cognitive decline in some populations.

## 1. Introduction

The increase in the numbers of people living into old age brings with it substantial challenges to global healthcare. Perhaps the most critical is the growing number of people living with dementia, currently estimated at around 50 million, with a new case every 3.2 s, and projected to double every two decades [1]. The relative lack of success of ‘single domain’ approaches to the disorder have led to the search for strategies which reduce the risk of dementia by delaying its onset and offsetting age-related cognitive decline. In this context, a number of lifestyle factors, including components of diet, have been identified as potential candidates for improving cognitive function. These include whole dietary patterns such as the Mediterranean diet and components of food such as the flavonoids [2].

Curcumin is a flavonoid, from the spice turmeric, with many characteristics that make it a candidate to improve physiological measures related to systemic and central health. Preclinical studies indicate that curcumin has properties relevant to neurocognitive functioning [3].

Regarding human health, there are several systemic processes which represent modifiable risk factors for cognitive decline [4]. Evidence from clinical trials suggest that many of these are positively modulated by curcumin in (despite a recent, widely publicised paper claiming, “No double blinded, placebo controlled clinical trial of curcumin has been successful”) [5]. For example a meta-analysis of randomised controlled trials (RCTs) of curcumin and tumour necrosis factor α (TNF α) levels, reported a significant reduction in this marker of inflammation across included studies [6]. Other RCTs have reported benefits to blood insulin (though not glucose) in healthy individuals [7], and improvements to several indices of glucoregulation in prediabetic and Type 2 diabetes [8]. Curcumin administration was associated with better cardiovascular function, increased central blood flow, decreased inflammation and oxidative stress, and qualitative changes to the gut microbiome.

Many of the same cardiometabolic processes are implicated in cognitive decline and dementia [4]. Thus, several placebo-controlled trials have investigated the potential for curcumin to improve cognitive function in both impaired and cognitively intact older cohorts. Small et al. [9] conducted an 18-month trial evaluating the efficacy of a bioavailable curcumin (180 mg of Theracurmin containing 10% curcumin). A cohort including both clinically intact individuals and those with Mild Cognitive Impairment were evaluated on standardised memory measures. A subset (15/arm) underwent [^18^F]FDDNP positron emission tomography (FDDNP-PET) to reveal amyloid and tau load (two key central markers of Alzheimer’s disease). Those in the curcumin group had significantly improved memory performance, along with reduced FDDNP-PET signal levels, indicating reduced amyloid and tau burden associated with curcumin treatment. This promising trial needs to be replicated with larger numbers.

Turning to effects of curcumin on cognition in healthy cohorts, Rainey-Smith and colleagues [10] conducted a 12-month trial of 1500 mg/day Biocurcumax™ in *n* = 96 healthy older adults (mean age, 66 years). Cognitive outcomes were measured at baseline, then at six and twelve months. Although there was a significant group x time interaction, the effects were not straightforward. Although the curcumin group outperformed placebo at six months, unfortunately the group scores were also significantly different at baseline, with an advantage for the placebo group, and statistically similar at the 12-month timepoint. This makes it difficult to disentangle the effects of the intervention from chance differences in performance.

Native curcumin has low bioavailability and likely has little efficacy unless large quantities are consumed daily. Several methods have been used to attempt to increase bioavailability. These include co-administering curcumin with piperine, or modifying the compound with lipidation. This is important since there appears to be a clear correspondence between curcumin bioavailability and its health benefits [11]. One solid-lipid curcumin preparation (SLCP), Longvida©, has been used in several preclinical and clinical trials. Primate studies indicate that Longvida© can protect against age-related cognitive decline [12].

Our previous RCT assessed the acute and chronic (28-day) mood and cognitive effects of 400 mg per day of Longvida© curcumin [13]. Compared with placebo, there were significant acute improvements to two measures of working memory, with one remaining significantly better at 28 days. Specifically, curcumin was associated with acutely improved Digit Vigilance and Serial Threes performance, and better Serial Threes performance following 28-day intervention. Additionally, there were a number of benefits to mood at 28 days. These included reduced self-rated fatigue as evaluated using the Chalder Fatigue Scale [14], a widely-used, 14-item scale gauging aspects of fatigue over the preceding week. At 28 days, there was also a significant attenuation of the negative effects of a cognitive challenge on alertness and contentment. Regarding biomarker assessment, curcumin was also associated with reduced total and low density lipoprotein [13].

These findings are supported by two recent systematic reviews (one including meta-analysis). The latter reported significant effects of curcumin over placebo, concluding that “curcumin is effective in improving memory performance in older adults” [15]. The other concluded that curcumin shows promise in improving cognitive function in both cognitively intact and compromised populations [16], but suggested more, well-designed studies are needed.

To summarise, there is accumulating evidence identifying curcumin as a candidate treatment to help prevent cognitive decline and dementia [17]. An obvious first step in evaluating this possibility is to test the efficacy to improve mood and cognition in healthy, older cohorts. In our previous study, this approach revealed significant improvements to mood and cognition in a 28-day intervention trial. Here we sought to extend the findings of that earlier trial by including a 12-week assessment in addition to a 28-day testing session.

The primary objective of this study was to evaluate whether the effects of our earlier 4-week trial could be replicated, and to extend the time course to evaluate if any effects were evident following 12-weeks curcumin supplementation. To this end, we conducted a double-blind RCT examining the effects of Longvida© curcumin on cognitive function, mood and wellbeing in healthy participants aged 50 to 85 years. We hypothesised that, compared with placebo, curcumin treatment would lead to reduced fatigue, improved working memory and attenuated stress reactivity.

## 2. Materials and Methods

This study was conducted in accordance with the guidelines of the Declaration of Helsinki. Ethical approval was provided by the Swinburne University Human Research Ethics Committee (SHR2016-008). The trial was registered on the Australian New Zealand Clinical Trials Registry as ACTRN12616000484448.

### 2.1. Design

The trial used a double-blind, placebo-controlled, parallel-groups design to investigate the effects on mood and cognitive function of 12-weeks supplementation with a bioavailability-enhanced curcumin extract, Longvida©, in healthy older adults. Specific biomarkers were also measured. An interim assessment was completed at 4-weeks. A subset of participants (target 50%) took part in a neuroimaging sub-study (the results of which will be published elsewhere).

### 2.2. Participants

**Figure 1:**
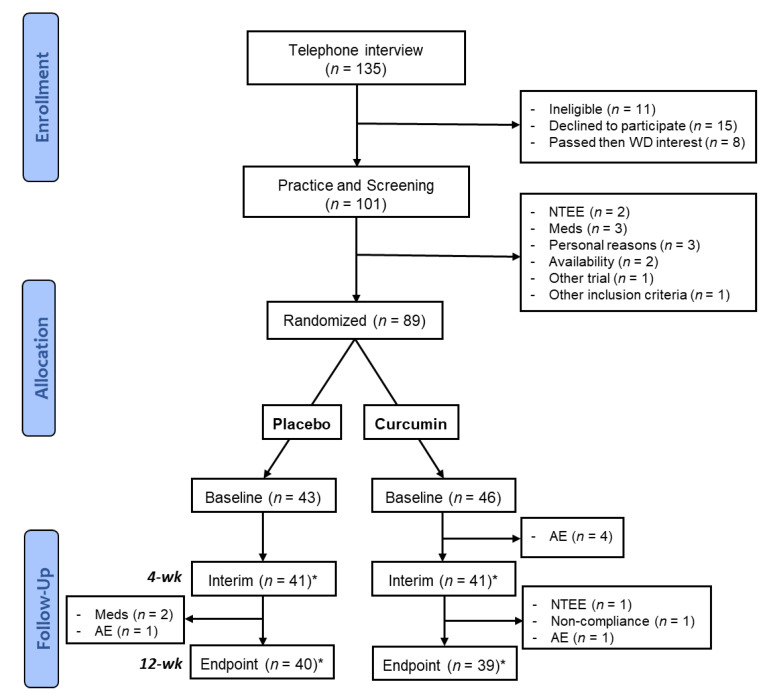
Modified Consort diagram showing participant recruitment and flow through the trial. Key: WD, withdrew; NTEE, Non-treatment emergent event (adverse event with onset prior to commencement of the trial intervention); Meds, use of medication listed in exclusion criteria; AE, adverse event; * Three participants (2 × Placebo, 1 × Curcumin) did not complete the Interim visit but did complete the Endpoint visit.

Figure 1 shows the recruitment pipeline. *n* = 79 participants completed the trial (from an initial target of 80). Participants who withdrew from the study prior to the final participant’s baseline visit were replaced to optimise the chance of meeting the target number of 80 active or completed participants.

Eligible participants were healthy men and women aged 50–85 years. Inclusion criteria included being free from medical conditions that may affect ability to participate in the study. That is no history of stroke, or neurological conditions (e.g., Parkinson’s, epilepsy), depression, psychiatric disorders, cognitive impairment, present or past alcohol abuse, being free from dementia, fluent in written and spoken English, having normal or corrected vision and not colour blind. Exclusion criteria included any significant concurrent illness including auto-immune disorder, bleeding disorders, currently impaired cardiovascular function, Type I diabetes, glaucoma, uncontrolled high blood pressure or gallstones or any known or suspected food allergies. Smokers and users of recreational drugs (except alcohol and other food grade actives) were excluded. Individuals who had participated in any other study involving an investigational product in the preceding 4-weeks were excluded, as were those taking anti-coagulant drugs, cholinergic drugs or steroid medications. Taking vitamins or herbal supplements reasonably suspected to influence study outcomes was also an exclusion criterion.

### 2.3. Treatment and Dosing Regime

The active treatment was 400 mg Longvida© Curcumin, consisting of approximately 80 mg curcumin in a SLCP formulation, with the remaining weight comprising of commonly used pharmaceutical excipients and small amounts of other curcuminoids present in turmeric extract. The placebo consisted of dextrin and a small amount of yellow food colouring, to match the appearance of curcumin supplements, smell and taste were also matched.

Treatment was administered orally as a single, once daily capsule taken between breakfast and lunch. The first dose was administered as part of the baseline visit with subsequent doses being self-administered by participants at home. The final dose was taken the day before the final assessment to ensure that follow-up assessments reflected the effects of chronic supplementation, rather than reflecting any superimposed, acute-on-chronic effects that may result from having curcumin or metabolites in circulation.

### 2.4. Randomisation and Treatment Allocation

In order to maintain blinding, randomisation and treatment allocation was carried out by a staff member external to the study. This individual was not involved in data collection or analysis. Treatment IDs were randomised 1:1 to active and control treatment. Randomisation was stratified by sex and MRI sub-study participation (to be reported elsewhere). Upon enrolment, participants were assigned the next consecutive three-digit ID number corresponding to a Treatment ID. Treatments were pre-packaged and labelled with Treatment ID, administration instructions and trial contact details. Treatment allocation remained the same throughout duration of the trial.

### 2.5. Treatment Compliance

Two methods were applied to monitoring treatment compliance: a count of returned treatments and a treatment log that was completed by participants at home. The pre-packaged, take-home treatments contained more capsules than required to maintain daily supplementation to trial completion. Participants were instructed to bring all unused capsules to their follow up visit/s, these were counted and percentage compliance with treatment was calculated as (number of capsules dispensed − number of capsules returned)/number of doses that should have been consumed (i.e., days since prior visit) × 100). In the event of a participant forgetting to return or losing capsules, the daily treatment log was used to estimate the number of capsules consumed. Participants who forgot to return their study treatment were provided with a prepaid envelope and asked to return all unused capsules as soon as possible. These were then used to confirm compliance, 80–120% compliance with treatment constituted eligibility to be included in analyses.

### 2.6. Recruitment

Participants were free-living older Australians, recruited using a variety of methods. Some were on the Centre for Human Psychopharmacology database of individuals who had previously requested to be informed about trial participation opportunities. Additionally, advertisements were placed in Seniors’ publications, at local clubs and organisations and on the University campus. All individuals who expressed interest in participation were provided with a copy of the Participant Information and Consent Form (PICF), which they were required to read before completing a formal Telephone Interview and being booked to enrol in the trial.

### 2.7. Procedure

Participants completed a telephone interview and four laboratory visits. The first of these was an enrolment, screening and practice visit. A baseline visit (Day 1) took place within 3 weeks of the practice visit, the 4-week visit at Day 28 (±4) and the 12-week visit at Day 84 (±4).

#### 2.7.1. Telephone Screening Interview

Prior to enrolment in the study, a structured Telephone Interview was completed to provide a preliminary eligibility assessment and to ensure the understanding of participation requirements. To verify the absence of dementia, the modified Telephone Interview for Cognitive Status (TICS-M) was completed [18]. The TICS-M assesses four domains of cognitive function: orientation, memory, attention/calculation and language, with the greatest weight placed on memory. Total scores range from 0 to 39, with larger scores indicative of superior cognitive function. There are no established cut-off points for the differentiation of cognitively intact, impaired or demented individuals using the 13-item TICS-m. Based on previous findings using this specific version [18,19], participants scoring less than 21 on the TICS-m would be excluded from the study on the grounds that moderate to severe cognitive impairment was indicated, suggesting the possibility of dementia.

#### 2.7.2. Enrolment, Screening and Practice Procedure

At this visit, written informed consent was obtained and eligibility was confirmed by a checklist and discussion of eligibility criteria. A medical history of any significant (i.e., lasting more than 2 weeks) illnesses or injuries occurring or treated within the past 5 years was taken and details of any concomitant medication use was recorded. While satisfaction of eligibility criteria was, in most cases by self-report, the Mini Mental State Exam (MMSE) [20] and Beck Depression Inventory (BDI-II) [21] were completed in order to screen for the presence of dementia and depression.

Demographic data were collected including date of birth, gender, dominant hand, whether English was their primary language, smoking history, highest level and total years of formal education and current employments status. Characterisation questionnaires were also completed (see respective trial measures for details). They were familiarised with study measures and completed practice versions of all cognitive assessments to minimise practice effects.

Volunteers were required to avoid significant changes to lifestyle factors such as habitual diet, exercise and medication (unless instructed by their doctor). In the event of such a change, they were asked to notify research staff to allow assessment of their ongoing trial eligibility.

#### 2.7.3. Study Visits

Participants arrived at the Centre for Human Psychopharmacology (CHP) in the morning between 8:30 a.m. and 10:30 a.m. On arrival, their compliance with having fasted from 10:00 p.m. the night before (consuming only water) and avoiding vigorous exercise were confirmed. A blood sample was collected and cardiovascular assessments were performed. They were provided with their chosen breakfast and, following a brief break, completed the chronic mood questionnaires and the computerised cognitive battery.

After completion of all mood and cognitive tests, participants were provided with a take home pack containing 32 treatment capsules, which was sufficient to allow once daily supplementation for the period from the Baseline to 4-week visit, plus additional capsules, a treatment log with instructions for completion, reminders of lifestyle and pre-visit restrictions (e.g., diet and exercise). They were administered their first study capsule prior to leaving the laboratory.

The 4-week visit followed the same procedure as the baseline visit except that take-home packs included 65 capsules and participants completed the Survey of Subjective Experience. The 12-week visit followed the same procedure as the baseline visit, except that no take home pack or treatment capsules were provided. Participants completed the Survey of Subjective Experience and were asked to indicate whether they believed that the treatment capsules they had been receiving were placebo or curcumin. At the conclusion of the visit, participants completed a reimbursement form.

Participants received 120 AUD at the end of their participation in the study, in the form of a cheque posted to their designated mailing address. Participants who completed the MRI sub-study received an additional 30 AUD (150 AUD total).

Once the trial was unblinded, participants were notified of their treatment allocation and those who had been in the placebo group were offered a free 12-week supply of Longvida© Curcumin. This ensured that all participants had equal opportunity to benefit from any positive effects of the treatment. The following sections detail the measures utilised in the study.

#### 2.7.4. Screening and Sample Characterisation

##### Mini Mental State Examination (MMSE)

The MMSE [20] was developed as a brief, quantitative assessment of cognitive function and is the most widely used measure for assessing cognitive status and detecting dementia in the elderly, both in research and clinical settings. The MMSE takes approximately 5–10 min to administer and assesses five domains of cognitive function: orientation, registration, attention/calculation memory and language. Participants scoring 20 or less, from a possible 30 were excluded from the study, as this is considered indicative of the presence of possible dementia.

##### Beck Depression Inventory-II (BDI-II)

The BDI-II [21] is one of the most widely used depression assessment tools in both clinical and research settings [22] and has been validated as measured for depression screening in community dwelling older adults [23]. It consists of 21 items to which participants respond according to how they have felt over the past two weeks. Each item is scored 0 to 3, resulting in possible total scores ranging from 0 to 63, with higher scores indicative of greater severity of depressive symptoms. A cut-off score of 20 was adopted for screening as this allows for the best detection of individuals with symptoms of moderate or severe depression [21].

##### Montreal Cognitive Assessment (MoCA)

The MoCA [24] measures global cognition and assesses cognitive domains of attention and concentration, executive function, memory, language, visuoconstructional skills, conceptual thinking, calculations and orientation. The MoCA was selected for the in-person characterisation of global cognitive function, over the MMSE, as it is less susceptible to a ceiling effect and has demonstrated superior sensitivity in the detection of early or mild cognitive impairment [24,25,26,27].

##### National Adult Reading Test (NART) 

The National Adult Reading Test (NART) [28] was used to characterise premorbid intelligence. It comprises an irregular word reading test that presents participants with 50 difficult or unusual words that they are required to read aloud. The number of words correctly pronounced is a widely-used estimate of premorbid Full-Scale IQ.

##### State-Trait Anxiety Inventory–Trait Scale (STAI-T) 

The State-Trait Anxiety Inventory (STAI) [29] comprises two scales. The ‘Trait’ scale (STAI-T) consists of 20 self-descriptive statements (e.g., “I feel pleasant”) to which participants respond by indicating how frequently each statement applies to how they “generally feel.” Total scores range from 20 to 80 with higher scores indicating greater dispositional anxiety.

#### 2.7.5. Chronic Mood and Wellbeing Questionnaires

The following questionnaires were completed at each assessment session (Baseline, 4-week and 12-week). They measured recent self-reported, state-non-specific feelings or experiences and were used to assess the effects of treatment on chronic mood and wellbeing. Items were administered in the following order.

##### Profile of Mood States (POMS) 

The POMS [30] consists of 65 mood-related adjectives to which participants respond by indicting to what extent they have been feeling that way over the past week on a 5-point scale from 0 (not at all) to 4 (extremely). Items are summed into six factors: Tension-Anxiety, Confusion-Bewilderment, Vigor-Activity, Anger-Hostility, Depression-Dejection and Fatigue-Inertia. A Total Mood Disturbance (TMD) score is computed as the sum of the first five factors minus Vigor-Activity. On all scales, except Vigor-Activity, which is reversed, a high score indicates greater mood disturbance.

##### Chalder Fatigue Scale (CFS)

The CFS [14] assesses the severity of fatigue experienced during the preceding week as compared to how one “usually” feels. It comprises 14 statements, six regarding symptoms of mental fatigue and eight regarding symptoms of physical fatigue, to which participants respond on a four-point scale (0 to 3), ranging from “less than usual” to “much more than usual.” Three resulting scores are calculated: Mental Fatigue, ranging from 0 to 18, Physical Fatigue ranging from 0 to 24 and Total Fatigue ranging from 0 to 42. Larger scores are indicative of greater fatigue.

##### Perceived Stress Scale (PSS) 

The PSS [31] consists of 14 items designed to measure a respondent’s perception of stress. Participants are asked to score on a scale from 0 to 4 how often they have felt a particular way over the past month. Total scores range from 0 to 56 with higher scores indicating a greater degree of perceived stress and lower scores indicating effective coping.

##### General Health Questionnaire (GHQ-28) 

The GHQ-28 [32] screens for symptoms on four subscales, each comprising seven items: somatic symptoms, anxiety/insomnia, social dysfunction and severe depression. Each item relates to complaints participants may have had “over the past few weeks,” to which they respond on a four point scale (0 to 3) indicating the extent to which they have been affected relative to how they usually feel, from “not at all” to “much more than usual.” Resulting scores on each subscale range from 0 to 21, and a total score from 0 to 84 is calculated.

##### Pittsburgh Sleep Quality Index (PSQI)

The PSQI [33] is a self-rated questionnaire that assesses sleep quality and disturbances over a 1-month time interval. Nineteen individual items generate seven “component” scores: subjective sleep quality, sleep latency, sleep duration, habitual sleep efficiency, sleep disturbances, use of sleeping medication and daytime dysfunction. The sum of scores for these seven components yields one global score.

##### Symptom Checklist

The symptom checklist comprised 28 items referring to possible mental or physical experiences such as “I have stomach pains,” “I have a change in energy” or “I feel stressed more than usual”. Participants were required to indicate to what extent the problem had distressed or bothered them in the past seven days, on a scale ranging from “not at all” to “very much so.” Responses to these items were used to examine any change in distressing symptoms that participants may have had following treatment administration and to assist in the detection of adverse events.

##### State Mood and Workload Assessment

These items measured the current, state-specific mood and were completed at each assessment session (Baseline, 4-weeks and 12-week), immediately before and after the cognitive battery. They were used to assess the perceived challenge posed by the cognitive battery and its effect on mood, and whether this could be modified by treatment. Specific items were as follows.

##### Visual Analogue Scales

The Bond-Lader Visual Analogue Scales [34] present participants with 16, 100 mm lines anchored on either end by opposing adjectives, such as “Alert” and “Drowsy.” For ease of use and improved accuracy over manual measurement, a computerised version of the Bond-Lader scale was used. Participants were presented with each line one at a time on the screen, and asked to use the mouse to place a repositionable X along the line according to how they felt at “this moment.” Participants were verbally reminded that the ends of the lines represented the very extremes of the named emotions. Responses were scored as percentage distance along the line from the negative anchor, providing a score from 0 to 100. The 16 items load onto three factors: Alertness, Calmness and Contentedness, with larger scores indicating higher levels of these moods.

In addition to the Bond-Lader scale, custom visual analogue scales were used to measure state stress, fatigue, anxiety and self-perceived ability to concentrate. For these items, participants were presented with a line anchored by “not at all” and “extremely” and asked to respond by indicating how they felt “at this moment.” The scales were scored as percentage distance from “Not at All” so that larger scores indicated greater levels of stress, fatigue and anxiety (undesirable), and better ability to concentrate (desirable).

##### Subjective Effects Questionnaire

In order to capture information about participants’ experiences, which may or may not have been detected by standardised questionnaires, three open ended questions were completed at the final study visit. These questions asked participants whether they had experienced any positive/negative/unusual (neither good nor bad) changes in their physical or mental health for which they did not know the cause or believed may be related to the study capsules. If yes, participants were asked to provide a brief written explanation.

##### NASA Task Load Index (NASA-TLX)

The NASA TLX [35] is a self-report measure of subjective workload. Participants are asked to rate the perceived mental, physical and temporal demands of a task from low to high, as well as how much effort was required (low to high), how they think they performed (good to bad) and how much frustration was caused (low to high). Participants are provided with the brief explanation of each scale, which ensures consistency of understanding and interpretation. As with the Visual Analogue Scales, the NASA-TLX items were each presented as single on-screen lines that were scored from 0 to 100.

#### 2.7.6. Computerised Cognitive Assessment

Standard cognitive tasks, with known psychometric properties, were presented using E-Prime 2.0 (PST Net) and Python 2.7.9. The battery took approximately 60 min to complete, and parallel versions of each task allowed a novel version for each testing session. Participants were provided with written instructions for each task and, during assessment, were monitored by a researcher who answered any questions and provided verbal explanation of the tasks if required.

##### Divided Attention Tracking Task (DATT)

The DATT task, a slight modification of that used by Scholey et al. [36], assessed the interfering effects of divided attention at the time of encoding on subsequent verbal recognition.

Participants were presented with 20 recordings of spoken words via headphones and asked to remember them. Each word was augmented to have a duration of exactly 1000 ms. In the Divided Attention Encoding condition, participants simultaneously used the computer mouse to track a moving on-screen dot. The dot moved in a random path at a rate of approximately 6 cm/sec. During Focused Attention Encoding, there was a tracking task.

In the subsequent Recognition phase, participants were presented with 80 spoken words via headphones: 40 new decoy words and 20 target words from each of the focused and divided attention conditions providing a 1:1 target to decoy ratio. Participants were asked to indicate whether they recognised each word as being one they heard earlier using keypad buttons labelled “Yes” and “No.” Participants were not required to distinguish the condition under which a word has been presented. A delay of approximately 30 min separated the encoding and recognition phases. In order to avoid differences in retention time being erroneously attributed to differences in encoding condition effects, the order of the Focused Attention Encoding and Divided Attention Encoding was counterbalanced across participants.

The task was scored for accuracy and speed of correct responses during the Recognition phase overall (correct recognition and correct rejection), correct recognition irrespective of encoding phase and separately for divided and focused encoding words. The key comparison was the difference between recognition accuracy for words presented in the divided and focused attention conditions.

##### Virtual Morris Water Maze (vMWM)

The Morris Water Maze (MWM) is a prototypical task for assessing spatial learning and memory in rodents [37]. Successful completion of the task requires that the subject learn the location of an escape platform submerged in a pool of cloudy water. In its original form, the animal uses distal cues to navigate to the previously learnt platform location.

In this virtual analogue of the task, participants are placed in a virtual room. They are required to navigate as quickly as possible to reach a platform hidden below the surface of the water and “escape from the pool.” The platform is in a fixed location, relative to environmental cues. The participant starts each trial at one of a number of different points around the outside of the pool. Outcome measures include time and length of path taken to reach the target; the initial heading errors are recorded for each trial, as well as time in target quadrant.

Participants began with four familiarisation trials in which the platform was visible and no cues were positioned in the room, and they were told that the visible platform location was no indication of where the hidden platform would be. Sixteen learning trials were then completed in which the platform was hidden below the surface of the water at the same location (no visible indication of its location was given). Participants started each trial facing the wall in 1 of 4 starting positions, trials were completed in 4 blocks, each comprising 1 trial at each of the starting locations, and outcomes were average within each block. They were instructed to make an active effort to find the platform even if they had no idea where it was (i.e., in the initial trials). If the platform was not found within 45 s, it became visible.

At the end of the 16 learning trials, a single immediate memory probe trial was completed in which, unbeknown to participants, the platform was absent. Participants started in a previously unused starting location, in the centre of a non-target quadrant, but were given no indication that this trial differed from the preceding 16. Successful learning would result in disproportionately greater time in the target quadrant. After a delay of approximately 30 min (during which other cognitive tasks were completed) participants were presented with a single delayed memory trial that was identical to the immediate memory probe. Again, time spent in the target quadrant was indicative of better spatial memory.

##### Serial Subtractions

A starting number between 800 and 999 was presented on screen. Participants were required to subtract three or seven (serial Threes and Serial Sevens, respectively) from this number and enter the three-digit response using the computer keyboard numeric keys. Each of the three-digit keystrokes was represented on-screen by an asterisk to ensure all calculations were performed mentally. After the first response was entered, the initial display was cleared from the screen and participants were required to continue subtracting three/seven from their previous answer. In case of an error, the accuracy of each response was scored with respect to the preceding response only. Participants were told to respond as quickly and accurately as possible and the total number of correct responses provided within three minutes was used as the measure of performance.

##### Arrow Flankers Task

This Go/No-Go modification of the Erikson Flanker Task [38] provided a measure of executive function and cognitive control, in particular interference suppression (the ability to ignore irrelevant or conflicting information) and response inhibition (the ability to refrain from providing an undesirable response). Similar modifications have demonstrated behavioural and neurological differences associated with cognitive maturation [39], cognitive impairment and dementia [40] and medication use in ADHD [41].

The task comprised four trial conditions. All conditions involved participants being presented with five on screen symbols in a horizontal line. The central “target” symbol was always an arrow pointing left or right, and participants were instructed to press the keyboard arrow key that corresponded to the direction of this target as quickly as possible. The “flanking” symbols varied by condition, but were always four of the same symbols: two on the left and two on the right of the target. Flanking symbols were either ‘facilitating’ (arrows pointing in the same direction as the target), ‘interfering’ (arrows pointing in the opposite direction to the target), ‘neutral’ (circles) or ‘no-go’ (Xs), where participants were instructed not to respond to the target as a measure of response inhibition.

#### 2.7.7. Biochemical Measures

All blood samples were collected via venipuncture. A range of analyses were performed to assess treatment safety and bioavailability and possible mechanisms of action. These included:

Potassium (mmol/L serum), Chloride (mmol/L serum), Bicarbonate (mmol/L serum), Urea (mmol/L serum), Creatinine (umol/L serum), eGFR (mL/min/1.73 m^2^), Urate (mmol/L serum), Calcium (mmol/L serum) and Phosphate (mmol/L serum).

Tests of liver function included total protein (g/L serum), Albumin (g/L serum), ALP (U/L serum), Bilirubin (umol/L serum), GGT (U/L serum), AST (U/L serum), ALT (U/L serum) and Glucose (mmol/L serum). Lipid profile measures included triglycerides (mmol/L serum), Total cholesterol (mmol/L serum), High density lipoprotein (HDL) (mmol/L serum), Low density lipoprotein (LDL) (mmol/L serum), LDL:HDL and Cholesterol:HDL.

Inflammatory markers, including highly sensitive C-reactive protein (hsCRP, mg/L serum), Estimated sedimentation rate (mm/h), Interleukin 1 Beta (IL-1β, pg/mL serum), Interleukin 6 (IL-6, pg/mL serum), Tumour necrosis factor alpha (TNFα, pg/mL serum), 8-hydroxy-2′ -deoxyguanosine (8-OHdG, ng/mL serum) and protein carbonyls (nmol/mg), were measured in house using commercially purchased ELISA kits as markers of oxidative stress.

#### 2.7.8. Statistical Analyses

Analyses were performed using IBM SPSS v25. Group characteristics were compared using one-way ANOVA or a non-parametric alternative according to data distribution. The planned method of analysis for investigation of treatment effects was Analysis of Covariance (ANCOVA) with post-treatment outcome as the dependent variable, baseline as a covariate and treatment as a between subjects factor. The 4-week and 12-week outcomes were assessed separately. Where the nature of the data did not permit the planned analyses, appropriate alternatives were used as indicated. Analyses of cognitive task performance, perceived demands and the effect of cognitive battery on state mood were repeated additionally, including the demographics variables age, years of education and cognitive status as measured by the TICS-M [18]. These variables were selected as covariates as they may have influenced how challenging participants found the cognitive battery. The TICS-M was chosen for inclusion as the measure of cognitive status, instead on the MoCA, for consistency with our previous study [13].

## 3. Results

In addition to the results presented below, detailed summary statistics of each outcome can be found in online Supplementary Material Tables S1–S7. Details of data transformations and statistical analyses are contained in online Supplementary Material Tables S8–S13.

### 3.1. Recruitment and Retention

A flow chart of participant recruitment and retention is shown in Figure 1 and detailed in Supplementary Material. Sample characteristics are presented in Table 1.

### 3.2. Treatment Compliance and Assessment Timing

All participants attending the 4-week visit satisfied the 80–120% compliance requirement for inclusion in analyses (mean 98.43%, ±4.06%). At the 12-week visit one participant reported having not taken their study capsules for 11 days, so was excluded from end-point analyses. The remaining participants all satisfied the compliance requirements (mean = 97.96%, SD = 3.61%). There was an average of 27.98 (±SD 1.28) days between baseline and 4-week assessment, and 83.78 (±1.47) between baseline and final assessment.

### 3.3. Description of Analysis Sample

The dataset used for analyses comprised of the participants for whom any post-treatment data were available and as a follow-up point of interest. This resulted in a total Baseline sample size of 85. These participants were aged 55.10 to 83.41 years (mean 68.10, SD 6.34 years), 49.4% were male and 90.6% were right-handed. Of the sample, 85.9% had undertaken tertiary or post-graduate education and an average of 16.36 years (SD 2.95 years) of formal education. A total of 57.6% of the sample were retired, 25.9% were working part time or casually and 14.1% were working full time. One participant reported that they were studying, and one classed themselves as unemployed. No participant was a current smoker, but 45.9% of participants reported that they had previously been a smoker. Time since quitting ranged from 2 to 60 years (mean 28.23, SD 14.06 years).

The baseline characteristics of the two treatment groups did not significantly differ in any demographic measured, nor in global cognitive function (MMSE, MoCA or TICS-M), estimate of “pre-morbid IQ” (NART), depressive symptoms (BDI-II) or trait anxiety (STAI-T). Further characteristics of the cohort can be found in the online Supplementary Material (Table 1 and Table S1).

Note that other effects, including on cognitive performance related to hippocampal function, which was also undertaken during neuroimaging, will be reported elsewhere.

### 3.4. Treatment Effects: Mood (See Also Supplementary Material Tables S2 and S3)

Significant effects and notable patterns in the data are presented below. Unless specified, these effects include adjustment for age, education and cognitive status. Details of relevant data transformations and more details on statistical analyses of each outcome can be found in the online Supplementary Material Table S2.

#### 3.4.1. Profile of Mood States (POMS)

**Figure 2:**
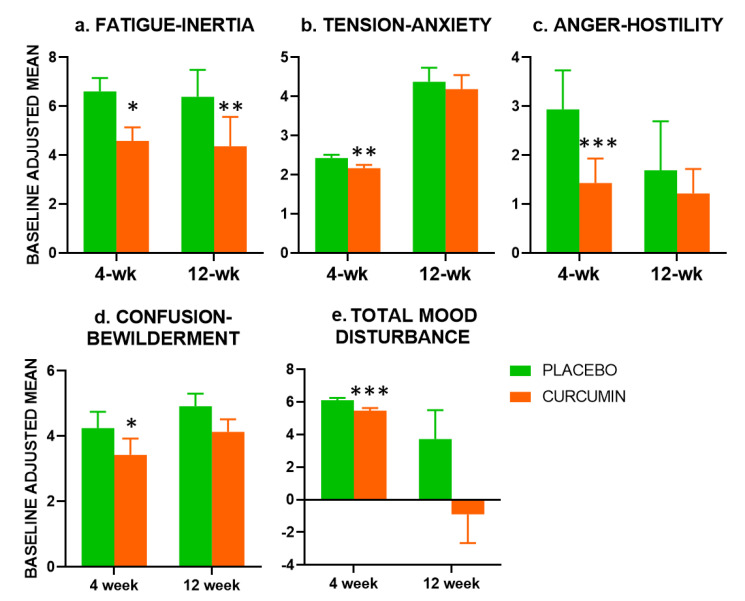
Effects of Longvida© curcumin on mood. Graphs depict baseline adjusted means with SEM (or 95% confidence intervals due to back transformation for a. 12-week, c. both, d. 4-week; see Supplementary Table S3 for unadjusted means and standard deviations). *, *p* < 0.05; **, *p* < 0.01; ***, *p* < 0.005.

Compared with placebo, the Fatigue-Inertia scale of the POMS was significantly improved in the curcumin group at both 4-week (F(1,79) = 6.795, *p* = 0.01) and 12-week (F(1,75) = 8.120, *p* = 0.006). Examination of the other subscales revealed significant beneficial effects of curcumin at 4-weeks only for Tension-Anxiety (F(1,77) = 8.073, *p* = 0.006), Confusion-Bewilderment (F(1,79) = 5.364, *p* = 0.023) and Anger-Hostility (F(1,78) = 8.952, *p* = 0.004). For Total Mood Disturbance (TMD) there was a significant beneficial effect of curcumin at 4-weeks (F(1,77) = 8.073, *p* = 0.006), but not 12-weeks. Effects on Depression-Dejection did not reach significance (*p* = 0.054), and Vigor-Activity did not show any evidence of a treatment effect (see Figure 2).

#### 3.4.2. NASA Task Load Index (NASA-TLX)

There were no significant differences between curcumin and placebo groups in the mental, physical or temporal demands of the cognitive battery perceived by participants in the at 4-week or 12-week assessments. At the 4-week assessment, participant in the curcumin group reported significantly lower ratings of their own performance (both with and without adjustment for age, education and cognitive status) (*p* < 0.05). At the same time, there were trends for the curcumin group to report lower levels of effort and frustration during the battery (*p* = 0.089 and *p* = 0.065, respectively). These differences did not survive adjustment for demographics.

At the 12-week analysis, there were no significant effects of treatment on perceived battery demands, or ratings of own performance, effort or frustration with or without adjustment for age, education and cognitive status.

#### 3.4.3. GHQ-28

There was no significant effect of treatment on scores on the Depression, Somatic Symptoms or Social Dysfunction subscales at either 4-week or 12-week assessments. There was a trend towards a benefit of curcumin treatment on change in Anxiety-Insomnia scale scores at the 4-week assessment (*p* = 0.056), but not at 12-week (*p* = 0.717).

#### 3.4.4. PSQI

There were no significant treatment effects on PSQI from analyses of variance. However, when data were analysed by chi-squared examining the direction of change, the curcumin group performed significantly better on the ‘daytime dysfunction’ subscale at 12-weeks (χ^2^ (n76, df2) = 8.019, *p* = 0.018).

There were no significant treatment effects on the CFS, or the PSS. Nor were there significant treatment effects at 4-week or 12-week on the change in calmness, alertness, contentedness, fatigue, stress, anxiety or ability to concentrate, induced by the performance of the cognitive battery.

When the analyses were repeated adjusting for participant age, education and cognitive status, the results were unchanged except that there was a non-significant trend (*p* = 0.083) for participants receiving placebo to have a lower increase in stress following the cognitive battery, at the 4-week assessment only.

### 3.5. Treatment Effects: Cognitive Function (See also Supplementary Material Tables S4 and S7)

#### 3.5.1. Divided Attention Tracking Task (DATT)

Analysis of task performance at baseline showed a highly significant (*p* < 0.001) effect of the secondary tasks on performance. Recognition accuracy for words presented during the divided attention (tracking) condition was significantly worse than during the focused condition. This demonstrates that the task manipulation had the planned effect with greater attentional load during encoding impairing later recognition.

There was no significant effect of treatment on overall recognition accuracy, recognition of words from either the divided attention or focused condition. For attentional load deficit (i.e., the difference in recognition of words presented in the divided and focused attention conditions), there was a marginal advantage for the curcumin group (F(1,78) = 3.982, *p* = 0.050) at the 12-week assessment only. This did not survive adjustment for age, education and cognitive status (F(1,72) = 3.640, *p* = 0.060).

#### 3.5.2. Virtual Morris Water Maze (vMWM)

**Figure 3:**
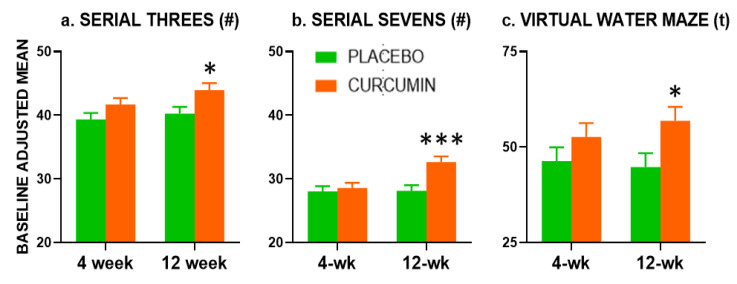
Positive effects of Longvida© on major cognitive outcomes. Graphs depict baseline adjusted means with Standard Error. Outcomes are number (#) correct for (**a**) Serial Threes and (**b**) Serial Sevens and time (t) in the target quadrant during the learning probe for (**c**) the virtual Morris Maze. *, *p* < 0.05; ***, *p* < 0.005.

At the 12-week assessment, there was a significant beneficial effect of treatment during learning probe performance, with participants in the curcumin group spending a higher proportion of time spent in the target quadrant (F(1,71) = 5.408, *p* = 0.023), suggesting greater learning and awareness of previous platform location (Figure 3c).

There was also a significant Trial × Treatment interaction that favoured the curcumin group (*p* = 0.019) at week 12. Further analyses of the block-by-block data revealed that from block 2 to block 3, participants in the placebo group showed an average decrease in performance, while participants in the curcumin group continued to improve. Participants in the curcumin group tended to spend more time in the target quadrant across all blocks. During block three, participants in the curcumin group spent significantly more time in the target quadrant (*p* = 0.007).

There was no significant effect of treatment on time spent in the target quadrant during the memory trial, at either the 4-week or 12-week assessments. These results were unchanged when age, education and cognitive status were included in the analyses.

#### 3.5.3. Serial Subtractions

Serial Subtractions results are presented in Figure 2a,b. At the 4-week assessment of the Serial Threes performance, there was a trend (*p* = 0.074) for participants receiving curcumin to provide a greater number of correct responses than those receiving placebo. This was no longer seen when controlling for age, education and cognitive status (*p* = 0.109). At the 12-week assessment, there was a significant beneficial effect of curcumin on the number of Serial Threes correct responses (F(1,72) = 5.764, *p* = 0.019).

At the 12-week assessment only, there was a significant (*p* = 0.001) beneficial effect of curcumin treatment on the Serial Sevens correct responses which remained significant when adjusting for age, education and cognitive status (F(1,72) = 12.837, *p* = 0.001).

#### 3.5.4. Arrow Flankers Tasks

Analysis of task performance at baseline showed a significant effect of congruence on Arrow Flankers reaction time. Response times in the congruent condition were significantly faster than in the neutral condition, which, in turn, were significantly faster than in the incongruent condition. This demonstrates that task manipulations had the planned effect, with the congruent condition facilitating responses and the incongruent condition causing interference.

At both the 4-week and 12-week assessments, there was no significant effect of treatment on overall reaction time across all conditions, or during the congruent, incongruent or neutral conditions. Nor was there any direct treatment effect on the interference effect of incongruent flankers (incongruent RT–neutral RT), or the faciliatory effect of congruent flankers (neutral RT–congruent RT).

Analysis of accuracy in no-go trials was not possible due to a ceiling effect in performance. A score of 90% or more was obtained by 74% of participants at baseline, 78% at the 4-week and 90.9% at the 12-week assessment. Instead, analysis was performed on the change in accuracy from baseline comparing the two treatment groups, and the two treatment groups were compared on the frequency of the direction of change, i.e., decline in accuracy, no change or improvements (Chi square). No significant effects of treatment were found.

The same Chi square analysis of accuracy across all conditions and within conditions found no significant effect of treatment at the 4-week analysis. There was a trend for a between groups difference of change in incongruent condition accuracy (*p* = 0.063).

At the 12-week analysis, the two groups showed a significantly different pattern of change in the faciliatory effect of congruent flankers, with the curcumin group being more likely to have an increase in faciliatory effect from the baseline to follow-up (χ^2^ (2, *n* = 73) = 7.612, *p* = 0.022).

### 3.6. Biochemical Measures

At the 12-week assessment, only participants in the curcumin arm had significantly higher levels of fasting glucose (mmol/L serum), with F(1,71) = 6.342, *p* = 0.014. The adjusted means for Placebo = 5.328 (Standard Error (SE) = 0.059), and the curcumin group = 5.542 (SE = 0.061).

There was no significant effect of treatment on any other tissue measure, i.e., renal function or electrolytes, liver function, lipid profile, cytokines, estimated sedimentation rate, c-reactive protein, DNA (8-OHdG) or protein (protein carbonyls) oxidative stress.

## 4. Discussion

As with our previous study [13], curcumin administration resulted in significant improvements to working memory and reductions in fatigue. These results support our hypothesis that the highly bioavailable curcumin preparation Longvida© can improve mood and cognition in older, cognitively intact people.

Our previous 4-week trial found improvements resulting from curcumin administration in Serial Threes performance at the 4-week endpoint [13]. In the study reported here, treatment effects on Serial Threes did not reach significance at 4-weeks (*p* = 0.074, 2-tailed), but were significant at 12-weeks. Similarly, significantly improved Serial Sevens performance emerged at the 12-week assessment only (see Figure 2). There were also benefits to vMWM performance at the 4-week timepoint.

There were no significant treatment effects on the Divided Attention task. Independent of condition, however, we did find the predicted differences in performance with respect to presence/absence of a secondary task during encoding. This suggests that participants were engaging with the task. Further research might usefully examine the effects of titrating cognitive load during multi-tasking to further explore the possibility of curcumin benefitting divided attention tasks.

Regarding the Arrow Flankers task, there were no significant direct effects of curcumin on performance. However, there did appear to be differential treatment effects with respect to congruence. Specifically, those in the curcumin arm appeared more sensitive to the facilitatory effect of congruent flankers. While it is difficult to draw conclusions from this single finding, it may reflect more cognitive resources being allocated to the learning of task strategies, an effect which is consistent with known physiological effects of curcumin.

Turning to mood effects, as in our previous study, curcumin administration led to reductions in fatigue at both 4 and 12-weeks. It should be noted that in this study, the reduction was in the POMS subscale of Fatigue-Inertia rather than the Chalder Fatigue Scale in our previous study [13]. Nevertheless, the results are broadly consistent across the two trials. Fatigue was a primary outcome in this trial (albeit measured using the CFS rather than the POMS). It is worth noting that in the current study, the 65-item POMS preceded the CFS. It may simply be that the first scale encountered is better placed to capture mood effects simply because participants engage more with earlier measures. The reduction in fatigue at both the 4-week and 12-week timepoints and in the (single post treatment) 4-week assessment in our previous study suggests that it is a robust effect of Longvida© curcumin in this population. This is also supported by the possibility of significant improvements in the ‘daytime dysfunction’ subscale of the PSQI, suggesting that the reductions in fatigue may have ramifications for everyday function. This finding should be explored in more detail, including utilising actigraphy to assess if fatigue reductions were manifesting in greater levels of activity.

Unlike our previous study, there were no effects of treatment on stress reactivity. That is, there were no treatment-related effects on mood changes occurring in response to completing the cognitive battery. The reasons for the slightly different pattern of the results may reflect methodological differences across the two studies. As an example, different mood scales were used. In study one, the Depression Anxiety and Stress Scale (DASS) was utilised [13,42]. In that study, the majority of participants scored zero on all three DASS scales, so here we instead opted to use the POMS. This revealed significant effects on a number of mood items. As well as reduced Fatigue-Inertia at both post-treatment assessments, the curcumin group had significant improvements in Tension-Anxiety, Confusion-Bewilderment and Anger-Hostility, as well as the overall Total Mood Disturbance scale at the 4-week assessment only. This suggests that a more comprehensive mood instrument may have higher utility in this kind of study.

Another methodological difference is that in our previous study [13], as well as at a 4-week assessment, participants were tested acutely 1 and 3 h following their first dose of curcumin. Acute-on-chronic effects were also evaluated by examining the same 1 and 3 h timepoints following the day-28 dose. Thus, the effects of treatment may have interacted with practice or repetition effects of cognitive testing. Significant benefits at 4-weeks in the previous study coincided with the third period of cognitive testing. Benefits seen here at 12-weeks would also represent the third testing event. This notion is supported to some extent by the modest facilitatory effect of congruent flankers in the curcumin group only, raising the possibility that curcumin facilitates learning. This possibility is also supported by the improvements to learning in the vMWM task. Future studies should also examine inter-individual differences in the bioavailability of curcumin (both the parent compound and its metabolites) and their relationship with changes in mood and cognition.

At the 12-week assessment only, participants in the curcumin arm had significantly higher blood glucose. It should be noted that this effect did not approach the levels to signal diabetes risk. In fact slightly elevated glucose levels are known to facilitate aspects of cognitive function including working memory and specifically Serial Sevens [43]. This suggests that this slight elevation in circulating glucose may contribute to the mechanisms of cognitive enhancement seen in the curcumin group. This possibility requires further exploration. On the other hand, it should be noted that the cholesterol reduction observed in our previous study was not replicated here, so the extent to which fluctuations in biomarkers are robust remains unknown.

The mechanisms by which the extract confers these mood and cognitive benefits are not known. They likely include multiple systemic and central mechanisms which have been ascribed to other nutrients [4], including flavonoids. Interestingly, the effects of other, more widely-researched compounds, e.g., cocoa flavanols, may be restricted to acute effects on mood and cognition [44], with longer term effects on mood alone [45].

While many tasks were not differentially significantly affected by treatment, it is worth noting that the vast majority of outcomes were numerically superior in the curcumin arm at both timepoints.

## 5. Conclusions

In conclusion, these data further support previous findings that Longvida© curcumin improves working memory and mood as well as the possibility of learning in healthy individuals. It is noteworthy that memory and fatigue are widely reported as the two more concerning non-physiological aspects of ageing [46]. This has potential promise to offset these effects and may also be relevant to conditions where mood and cognition are fragile.

## Figures and Tables

**Table 1:** Baseline Group Demographics. Except for gender, numbers are mean with standard deviation (SD) in brackets.

Variables	*Placebo (n = 43)*	*Curcumin (n = 42)*
% Male	48.84	50
Age (years)	68.38 (6.71)	67.81 (6.00)
TICS-M	28.09 (3.146)	27.79 (2.90)
MMSE	29.35 (0.94)	29.143 (1.095)
Years education	16.56 (3.048)	16.162 (2.879)
STAI-T	29.63 (7.41)	27.57 (5.739)
BDI	3.79 (4.29)	2.98 (3.37)
NART	40.465 (5.00)	40.48 (4.63)

**Key**. TICS-M = Telephone Interview for Cognitive Status, MMSE = Mini Mental State Exam, STAI-T = State-Trait Anxiety Inventory–Trait Scale, BDI = Beck Depression Inventory-II, NART = National Adult Reading Test.

## Supplementary Materials

The following are available online at https://www.mdpi.com/2072-6643/12/6/1678/s1, Table S1. Extended Baseline Group Demographics; Table S2. Change in state mood induced by performance of cognitive battery; Table S3. Chronic mood and wellbeing questionnaire scores; Table S4. Cognitive Battery Results; Table S5. NASA Task Load Index–Subjective Appraisal of Cognitive Battery; Table S6. Physiological measures; Table S7. Medians and interquartile ranges; Table S8. Unadjusted cognitive outcomes analysis; Table S9. Cognitive outcomes analysis adjusted for age, education and cognitive status; Table S10. Arrow Flankers chi-squared analysis; Table S11. Analysis of mood outcomes; Table S12. Categorical Analysis of PSQI; Table S13. Biomarker Analysis.
