# Supplementary material for: Further Evidence of Benefits to Mood and Working Memory from Lipidated Curcumin in Healthy Older People: A 12-Week, Double-Blind, Placebo-Controlled, Partial Replication Study
Source: Nutrients. 2020 Jun 4;12(6):1678. doi: 10.3390/nu12061678 (PMC7352411; doi:10.3390/nu12061678)
Supplement: Supplementary file 1 [file nutrients-12-01678-s001.zip › nutrients-811597-supplementary.pdf]

SUPPLEMENTARY TABLES: notes

- Numbers vary between visits and measures due to missing data or outliers that have been removed.
- For variables highlighted in blue the data for placebo and/or curcumin were skewed. Medians and IQRs for these measures are presented in Table S7.
- Summary statistics for all visits are included, even if only one visit's data were skewed to allow meaningful comparison.

Table S1. Extended Baseline Group Demographics

	Placebo (n = 43)		Curcumin (n = 42)	
	Mean	sd	Mean	sd
Age	68.38	6.712	67.811	6.002
TICS-M	28.093	3.146	27.786	2.901
MMSE	29.349	0.948	29.143	1.095
EduYears	16.558	3.048	16.162	2.879
STAI-T	29.628	7.41	27.571	5.739
BDI	3.791	4.285	2.976	3.368
NART	40.465	4.997	40.476	4.629
Male	48.84%		50.00%	
English primary Language	100.00%		97.62%	
Right handed	90.70%		90.48%	
Previous smoker	48.84%		42.86%	
Education level				
Primary	0.00%		2.38%	
Secondary	13.95%		11.90%	
Tertiary	58.14%		64.29%	
Postgraduate	27.91%		21.43%	
Employment				
Retired	58.14%		57.14%	
Unemployed	2.33%		0.00%	
Part Time/Casual	30.23%		21.43%	
Studying	0.00%		2.38%	
Full Time	9.30%		19.05%	

Table S2. Change in state mood induced by performance of cognitive battery

	Placebo			Curcumin		
	n	Mean	sd	n	Mean	sd
**Calmness**						
Baseline	43	-6.953	12.587	41	-4.256	12.110
Interim (4 weeks)	41	-6.683	14.854	40	-7.588	14.496
Endpoint (12 weeks)	40	-5.863	12.261	37	-7.230	14.129
**Alertness**						
Baseline	43	-12.424	8.407	42	-13.130	11.285
Interim (4 weeks)	41	-12.222	11.231	41	-10.089	13.012
Endpoint (12 weeks)	40	-11.133	11.052	37	-9.937	10.006
**Contentedness**						
Baseline	43	-6.674	10.563	42	-5.529	9.270
Interim (4 weeks)	41	-5.473	11.082	41	-6.195	14.132
Endpoint (12 weeks)	40	-5.830	9.691	38	-2.921	10.612
**Stress**						
Baseline	43	28.465	33.452	42	32.952	34.233
Interim (4 weeks)	41	30.707	30.112	41	40.073	30.828
Endpoint (12 weeks)	40	36.700	27.535	38	36.158	30.603
**Anxiety**						
Baseline	43	8.907	16.064	42	5.714	20.977
Interim (4 weeks)	41	4.390	15.328	41	7.000	18.473
Endpoint (12 weeks)	39	6.897	14.549	38	3.868	17.802
**Fatigue**						
Baseline	43	10.744	15.189	41	15.829	17.832
Interim (4 weeks)	41	9.024	20.597	41	3.268	19.320
Endpoint (12 weeks)	40	5.650	18.777	38	3.000	24.787
**Ability to concentrate**						
Baseline	43	-34.488	29.427	42	-45.571	28.910
Interim (4 weeks)	41	-30.683	32.545	41	-38.951	32.778
Endpoint (12 weeks)	40	-40.300	29.480	38	-39.684	28.887

Table S3. Chronic mood and wellbeing questionnaire scores

	Placebo			Curcumin		
	n	Mean	sd	n	Mean	sd
**CHALDER FATIGUE SCALE**						
**Total Fatigue**						
Baseline	42	14.262	3.575	42	14.595	2.846
Interim (4 weeks)	41	14.317	3.350	41	14.122	3.763
Endpoint (12 weeks)	39	15.256	4.140	39	14.308	4.420
**Physical Fatigue**						
Baseline	42	8.214	2.203	42	8.476	2.051
Interim (4 weeks)	41	8.341	2.287	41	8.122	2.451
Endpoint (12 weeks)	39	8.974	2.870	39	8.359	2.861
**Mental Fatigue**						
Baseline	42	6.048	1.780	42	6.119	1.087
Interim (4 weeks)	41	5.976	1.508	41	6.000	1.517
Endpoint (12 weeks)	39	6.282	1.806	39	5.949	1.835
**PROFILE OF MOOD STATES (POMS)**						
**Total Mood Disturbance (TMD)**						
Baseline	43	4.395	19.786	41	2.146	19.351
Interim (4 weeks)	40	7.725	19.049	41	-0.659	18.578
Endpoint (12 weeks)	39	4.077	14.414	39	-0.538	14.807
**Tension-Anxiety**						
Baseline	43	5.442	3.354	41	4.902	3.338
Interim (4 weeks)	40	5.575	3.587	41	4.122	3.219
Endpoint (12 weeks)	39	4.513	2.553	39	4.051	2.733
**Depression-Dejection**						
Baseline	43	3.256	4.271	41	3.098	5.142
Interim (4 weeks)	40	3.950	3.883	41	2.976	3.725
Endpoint (12 weeks)	39	2.692	2.939	39	2.564	3.455
**Anger-Hostility**						
Baseline	43	3.884	4.119	41	3.220	3.029
Interim (4 weeks)	41	4.585	4.582	41	2.659	3.832
Endpoint (12 weeks)	39	2.564	2.808	39	2.051	2.575
**Confusion-Bewilderment**						
Baseline	43	5.093	3.753	42	4.833	3.378
Interim (4 weeks)	41	5.415	3.808	41	4.049	2.792
Endpoint (12 weeks)	39	5.000	3.253	39	4.051	2.762
**Fatigue-Inertia**						
Baseline	43	5.535	5.404	42	5.667	3.874
Interim (4 weeks)	41	6.585	5.277	41	4.610	4.030

Endpoint (12 weeks)	39	7.026	4.374	39	5.000	3.811
**Vigor-Activity**						
Baseline	43	18.814	4.742	42	19.167	6.378
Interim (4 weeks)	41	17.610	4.852	41	19.073	5.561
Endpoint (12 weeks)	39	17.718	4.039	39	18.256	4.935
**PERCEIVED STRESS SCALE (PSS)**						
Baseline	42	10.857	4.657	42	11.310	5.106
Interim (4 weeks)	41	11.268	5.613	41	11.634	5.634
Endpoint (12 weeks)	39	10.231	4.445	39	11.718	5.341
**GENERAL HEALTH QUESTIONNAIRE (GHQ-28)**						
**Total**						
Baseline	43	15.419	5.687	42	16.048	7.054
Interim (4 weeks)	41	15.732	6.637	41	15.146	8.104
Endpoint (12 weeks)	39	15.436	5.661	39	15.872	10.222
**Somatic Symptoms**						
Baseline	43	4.186	2.822	42	4.214	3.041
Interim (4 weeks)	41	4.220	3.103	41	4.463	3.302
Endpoint (12 weeks)	39	4.769	3.391	39	4.615	3.603
**Anxiety-Insomnia**						
Baseline	43	3.907	2.724	42	4.167	2.775
Interim (4 weeks)	41	4.537	3.279	41	3.366	2.809
Endpoint (12 weeks)	39	3.410	2.161	39	3.667	3.223
**Social Dysfunction**						
Baseline	43	6.837	1.526	42	7.048	1.937
Interim (4 weeks)	41	6.659	1.811	41	6.561	1.845
Endpoint (12 weeks)	39	6.872	1.542	39	6.615	2.347
**Depression**						
Baseline	43	0.488	0.736	42	0.619	1.324
Interim (4 weeks)	41	0.317	0.850	40	0.475	1.062
Endpoint (12 weeks)	39	0.385	0.907	39	0.974	2.758
**PITTSBURGH SLEEP QUALITY INDEX (PSQI)**						
**Global Score**						
Baseline	41	6.146	3.403	40	5.350	2.694
Interim (4 weeks)	40	6.025	3.230	41	5.073	2.659
Endpoint (12 weeks)	39	6.179	2.981	39	5.667	2.629
**Subjective Sleep Quality**						
Baseline	43	0.907	0.570	41	0.829	0.667
Interim (4 weeks)	40	0.900	0.632	41	0.780	0.652
Endpoint (12 weeks)	39	0.821	0.644	39	0.897	0.641

**Sleep Latency**						
Baseline	43	1.000	0.976	41	0.683	0.722
Interim (4 weeks)	41	0.951	0.865	41	0.537	0.674
Endpoint (12 weeks)	39	0.923	0.870	39	0.795	0.732
**Sleep Duration**						
Baseline	43	0.395	0.695	41	0.293	0.559
Interim (4 weeks)	41	0.366	0.623	41	0.293	0.559
Endpoint (12 weeks)	39	0.385	0.590	39	0.282	0.510
**Sleep Efficacy**						
Baseline	43	1.256	1.311	42	0.881	1.173
Interim (4 weeks)	41	1.220	1.314	41	0.902	1.020
Endpoint (12 weeks)	39	1.179	1.144	39	0.974	1.135
**Sleep Disturbance**						
Baseline	43	1.326	0.474	41	1.293	0.461
Interim (4 weeks)	41	1.268	0.549	41	1.317	0.471
Endpoint (12 weeks)	39	1.308	0.468	39	1.385	0.544
**Use of Medication**						
Baseline	43	0.256	0.759	41	0.146	0.478
Interim (4 weeks)	41	0.195	0.641	41	0.098	0.374
Endpoint (12 weeks)	39	0.282	0.759	39	0.154	0.432
**Daytime Dysfunction**						
Baseline	41	1.122	0.331	40	1.175	0.446
Interim (4 weeks)	41	1.268	0.549	41	1.146	0.422
Endpoint (12 weeks)	39	1.282	0.456	39	1.179	0.389

Table S4. Cognitive Battery Results

	Placebo			Curcumin		
	n	Mean	sd	n	Mean	sd
**VIRTUAL MORRIS WATER MAZE**						
**Time in Target Quadrant During Learning Trials**						
Baseline						
Block 1	42	44.475	15.733	42	43.722	15.460
Block 2	42	49.435	19.605	42	55.885	19.691
Block 3	42	51.651	15.735	42	55.004	19.210
Block 4	42	48.656	17.155	42	56.584	18.118
Interim (4 weeks)						
Block 1	41	43.604	13.021	41	46.324	12.896
Block 2	41	53.337	19.015	41	53.029	16.879
Block 3	41	44.486	18.593	41	55.356	16.880
Block 4	41	47.586	18.699	41	54.106	17.504
Endpoint (12 weeks)						
Block 1	40	46.959	14.555	38	52.081	13.891
Block 2	40	55.237	20.294	38	61.771	19.055
Block 3	40	51.509	16.196	38	58.304	18.443
Block 4	40	52.619	17.184	38	56.807	17.523
**Time in Target Quadrant During Immediate Learning Probe**						
Baseline	42	45.702	29.317	42	43.728	26.017
Interim (4 weeks)	41	47.367	24.587	41	51.662	27.418
Endpoint (12 weeks)	40	44.323	27.018	38	57.222	22.374
**Time in Target Quadrant During Delayed Memory Probe**						
Baseline	42	52.029	25.220	42	48.841	26.880
Interim (4 weeks)	41	43.660	23.441	41	50.246	26.723
Endpoint (12 weeks)	40	46.643	28.287	38	54.062	24.064
**DIVIDED ATTENTION TASK**						
**Overall Recognition Accuracy**						
Baseline	43	69.126	8.741	42	68.148	7.853
Interim (4 weeks)	41	71.032	7.687	41	69.902	7.625
Endpoint (12 weeks)	40	70.870	8.723	38	70.747	7.899
**Focused Attention Encoding Recognition Accuracy**						
Baseline	43	63.372	17.584	42	59.762	17.142
Interim (4 weeks)	41	69.146	16.121	41	63.537	19.819
Endpoint (12 weeks)	40	69.875	17.227	38	61.842	18.651

**Divided Attention Encoding Recognition Accuracy**						
Baseline	43	57.907	19.525	42	52.381	16.463
Interim (4 weeks)	41	56.341	17.784	41	53.537	17.827
Endpoint (12 weeks)	40	53.500	18.852	38	55.000	18.744
**Divided Attention Accuracy Deficit[1]**						
Baseline	43	-5.465	14.426	42	-7.381	17.188
Interim (4 weeks)	41	-12.805	16.585	41	-10.000	22.333
Endpoint (12 weeks)	40	-16.375	21.779	38	-6.842	19.879
**Overall Correct Response Time**						
Baseline	43	1562.000	267.757	42	1537.976	201.178
Interim (4 weeks)	41	1557.488	254.225	41	1508.683	208.614
Endpoint (12 weeks)	40	1543.850	223.727	38	1527.105	205.992
**Focused Attention Encoding Recognition Response Time**						
Baseline	43	1522.070	251.766	42	1502.357	194.459
Interim (4 weeks)	41	1518.171	240.522	41	1462.024	179.152
Endpoint (12 weeks)	40	1481.275	203.691	38	1492.500	188.378
**Divided Attention Encoding Recognition Response Time**						
Baseline	43	1563.070	263.827	42	1585.143	248.372
Interim (4 weeks)	41	1562.829	247.423	40	1529.425	224.407
Endpoint (12 weeks)	40	1582.900	275.147	38	1550.026	221.567
**Divided Attention Response Time Deficit[2]**						
Baseline	43	41.000	140.582	42	82.786	154.637
Interim (4 weeks)	41	44.659	137.976	39	44.231	111.542
Endpoint (12 weeks)	40	101.625	196.371	38	57.526	132.567
**SERIAL SUBTRACTION TASK Serial 3 Subtraction, Number of Correct Responses**						
Baseline	43	39.837	15.816	42	39.381	13.949
Interim (4 weeks)	41	39.439	17.762	41	41.585	13.733
Endpoint (12 weeks)	40	40.175	14.672	38	44.026	12.536
**Serial 7 Subtraction, Number of Correct Responses**						
Baseline	43	27.581	12.743	42	27.738	12.688
Interim (4 weeks)	41	28.000	13.524	41	28.610	11.526
Endpoint (12 weeks)	40	27.850	13.235	38	32.947	11.905

**ARROW FLANKERS TASK**						
**Overall Accuracy**						
Baseline	43	95.659	7.031	42	96.567	4.638
Interim (4 weeks)	41	97.358	4.039	41	97.216	3.889
Endpoint (12 weeks)	40	97.625	3.239	38	96.009	6.261
**Congruent Flankers Condition Accuracy**						
Baseline	43	98.061	2.813	42	97.420	4.483
Interim (4 weeks)	41	97.967	3.929	41	97.967	3.780
Endpoint (12 weeks)	40	97.846	3.299	38	98.318	3.265
**Incongruent Flankers Condition Accuracy**						
Baseline	43	97.802	3.005	41	97.222	4.521
Interim (4 weeks)	41	97.221	5.625	41	97.425	4.230
Endpoint (12 weeks)	40	97.083	5.832	38	96.125	7.765
**Neutral Flankers Condition Accuracy**						
Baseline	41	98.103	3.133	42	98.214	4.107
Interim (4 weeks)	41	97.899	4.015	41	97.696	4.344
Endpoint (12 weeks)	40	98.263	3.062	37	97.597	4.950
**No-Go Flankers Condition Accuracy**						
Baseline	41	92.480	9.092	42	90.874	8.192
Interim (4 weeks)	41	94.309	9.938	41	92.887	8.653
Endpoint (12 weeks)	40	96.668	6.201	37	93.470	6.253
**Accuracy During Congruent vs Neutral Conditions (Faciliatory Effect)[3]**						
Baseline	41	0.000	2.635	42	-0.794	2.899
Interim (4 weeks)	41	0.068	3.010	41	0.271	2.835
Endpoint (12 weeks)	40	-0.417	3.302	37	0.675	4.213
**Accuracy During Incongruent vs Neutral Conditions (Interference Effect)[4]**						
Baseline	41	-0.204	2.873	41	-0.948	3.547
Interim (4 weeks)	41	-0.678	4.016	41	-0.271	2.548
Endpoint (12 weeks)	40	-1.181	6.029	37	-1.577	5.302
**Overall Response Time of Correct Responses**						
Baseline	43	594.233	62.865	42	595.833	64.905
Interim (4 weeks)	41	596.951	73.289	41	588.390	63.457
Endpoint (12 weeks)	40	595.450	71.745	38	588.184	59.200

**Correct Response Time During Congruent Condition**						
Baseline	43	577.814	57.814	42	582.905	65.292
Interim (4 weeks)	41	582.463	70.483	41	574.390	58.579
Endpoint (12 weeks)	40	577.900	71.310	38	573.395	58.983
**Correct Response Time During Incongruent Condition**						
Baseline	43	618.186	70.968	42	616.524	68.163
Interim (4 weeks)	41	614.902	74.301	41	607.976	67.364
Endpoint (12 weeks)	40	620.675	76.732	38	608.895	63.980
**Correct Response Time During Neutral Condition**						
Baseline	41	582.390	63.828	42	588.786	66.729
Interim (4 weeks)	41	593.561	79.450	41	582.902	68.403
Endpoint (12 weeks)	40	588.550	72.482	37	582.108	61.168
**Correct Response Time During Congruent vs Neutral Conditions (Faciliatory Effect)[5]**						
Baseline	41	-6.878	21.742	42	-5.881	23.175
Interim (4 weeks)	41	-11.098	25.314	41	-8.512	24.764
Endpoint (12 weeks)	40	-10.650	22.258	37	-9.459	20.382
**Correct Response Time During Incongruent vs Neutral Conditions (Interference Effect)[6]**						
Baseline	41	29.805	29.748	42	27.738	28.084
Interim (4 weeks)	41	21.341	23.899	41	25.073	23.756
Endpoint (12 weeks)	40	32.125	27.863	37	25.459	28.758

[1] Calculated as recognition accuracy for divided attention words - recognition accuracy for focused attention words. Negative scores indicate poorer accuracy due to divided attention.

[2] Calculated as response time for focused attention words - response time for divided attention words. Positive scores indicate slower response due to divided attention.

[3] Calculated as accuracy during congruent trials - accuracy during neutral trials. Positive scores indicate better accuracy in the presence of congruent flankers.

[4] Calculated as accuracy during incongruent trials - accuracy during neutral trials. Negative scores indicate poorer accuracy in the presence of incongruent flankers.

[5] Calculated as correct response time during congruent trials - correct response time during neutral trials. Negative scores indicate faster responses in the presence of congruent flankers.

[6] Calculated as correct response time during incongruent trials - correct response time during neutral trials. Positive scores indicate slower responses in the presence of incongruent flankers.

Table S5. NASA Task Load Index – Subjective Appraisal of Cognitive Battery

	Placebo			Curcumin		Placebo	
	n	Mean	sd		n	Mean	sd
**Mental Demand**							
Baseline	43	68.093	17.490	41	72.244	2.400	15.368
Interim (4 weeks)	41	70.171	15.043	40	72.425	1.803	11.406
Endpoint (12 weeks)	40	68.525	15.828	38	68.316	2.519	15.530
**Temporal Demand**							
Baseline	43	59.000	16.248	42	55.667	2.613	16.937
Interim (4 weeks)	41	57.439	18.231	41	56.293	3.189	20.422
Endpoint (12 weeks)	40	56.850	20.322	38	55.026	3.157	19.460
**Physical Demand**							
Baseline	43	28.000	18.431	42	26.095	2.824	18.301
Interim (4 weeks)	41	28.390	23.484	41	29.683	3.262	20.887
Endpoint (12 weeks)	40	28.000	21.775	38	27.842	3.145	19.384
**Own Performance**							
Baseline	43	44.698	23.403	42	43.262	3.158	20.463
Interim (4 weeks)	41	44.317	22.366	41	36.049	2.844	18.210
Endpoint (12 weeks)	40	44.475	21.770	38	36.526	3.010	18.557
**Effort**							
Baseline	43	67.628	14.844	42	69.381	2.563	16.613
Interim (4 weeks)	41	70.171	12.667	41	66.439	2.637	16.882
Endpoint (12 weeks)	39	66.513	16.065	38	64.342	2.782	17.152
**Frustration**							
Baseline	43	44.488	23.719	42	42.143	3.455	22.394
Interim (4 weeks)	41	52.293	23.579	41	42.805	3.586	22.964
Endpoint (12 weeks)	40	44.825	23.132	38	37.474	3.616	22.291

Table S6. Physiological measures

		Placebo			Curcumin		
		n	Mean	sd	n	Mean	sd
**Renal and Liver Function**							
Sodium (mmol/L)	Baseline	43	141.860	2.178	42	141.381	1.987
	Endpoint (12 weeks)	39	141.436	1.759	37	141.514	1.909
Potassium (mmol/L)	Baseline	43	4.530	0.290	42	4.483	0.348
	Endpoint (12 weeks)	39	4.415	0.300	37	4.478	0.350
Chloride (mmol/L)	Baseline	43	106.279	1.919	42	106.000	1.767
	Endpoint (12 weeks)	39	105.923	1.612	37	106.081	1.963
Bicarbonate (mmol/L)	Baseline	43	30.721	1.968	42	30.524	2.144
	Endpoint (12 weeks)	39	30.231	2.311	37	30.189	1.956
Urea (mmol/L)	Baseline	43	6.247	2.020	42	5.867	1.656
	Endpoint (12 weeks)	39	5.882	1.459	37	5.757	1.272
Creatinine (µmol/L)	Baseline	43	79.256	13.206	42	77.524	16.959
	Endpoint (12 weeks)	39	78.308	11.855	37	80.730	15.064
UricAcid (mmol/L)	Baseline	43	0.339	0.087	42	0.321	0.104
	Endpoint (12 weeks)	39	0.334	0.089	37	0.321	0.095
Calcium (mmol/L)	Baseline	43	2.352	0.076	42	2.309	0.094
	Endpoint (12 weeks)	39	2.327	0.067	37	2.331	0.088
ljusted Calcium (mmol/L)	Baseline	43	2.375	0.078	42	2.335	0.092
	Endpoint (12 weeks)	39	2.348	0.055	37	2.348	0.083
Phosphate (mmol/L)	Baseline	43	1.092	0.156	42	1.128	0.126
	Endpoint (12 weeks)	39	1.122	0.151	37	1.139	0.154
T.Protein (g/L)	Baseline	43	69.163	3.970	42	69.119	4.702
	Endpoint (12 weeks)	39	68.436	3.662	37	68.568	4.381
Albumin (g/L)	Baseline	43	38.605	1.978	42	38.429	2.154
	Endpoint (12 weeks)	39	38.846	1.981	37	39.108	1.792
ALP (U/L)	Baseline	43	72.186	16.796	42	70.119	18.075
	Endpoint (12 weeks)	39	70.590	16.671	37	65.108	16.017
Bilirubin (µmol/L)	Baseline	43	10.907	3.822	42	12.429	5.438
	Endpoint (12 weeks)	39	10.769	3.383	37	12.405	5.241
GGT (U/L)	Baseline	41	23.268	15.022	42	21.476	10.703
	Endpoint (12 weeks)	37	23.892	16.571	37	19.973	12.036
AST (U/L)	Baseline	43	20.977	5.054	42	20.929	3.023
	Endpoint (12 weeks)	38	20.921	4.327	36	21.917	4.519
ALT (U/L)	Baseline	41	20.220	7.904	42	18.071	6.256
	Endpoint (12 weeks)	37	20.568	8.792	36	20.278	8.879
Glucose (mmol/L)	Baseline	42	5.507	0.778	42	5.400	0.868
	Endpoint (12 weeks)	39	5.490	1.249	37	5.584	1.156
**Lipids**							
Triglycerides (mmol/L)	Baseline	42	1.257	0.612	42	1.157	0.629
	Endpoint (12 weeks)	38	1.216	0.687	37	1.170	0.620
Total_Chol (mmol/L)	Baseline	43	5.216	1.064	42	5.095	1.297
	Endpoint (12 weeks)	39	5.226	0.959	37	5.038	1.207
HDL_Chol (mmol/L)	Baseline	43	1.584	0.428	42	1.731	0.578
	Endpoint (12 weeks)	38	1.605	0.400	37	1.657	0.540

LDL_Chol (mmol/L)	Baseline	43	3.070	0.939	42	2.879	0.926
	Endpoint (12 weeks)	38	3.050	0.879	37	2.884	0.870
NonHDL_Chol (mmol/L)	Baseline	43	3.656	0.926	42	3.383	1.008
	Endpoint (12 weeks)	38	3.632	0.872	37	3.405	1.019
LDLHDL_Ratio	Baseline	43	2.084	0.678	41	1.766	0.592
	Endpoint (12 weeks)	38	2.037	0.692	36	1.844	0.627
CholHDL_Ratio	Baseline	43	3.477	0.783	41	3.085	0.763
	Endpoint (12 weeks)	38	3.434	0.796	36	3.186	0.817
**Inflammation**							
hsCRP (mg/L)	Baseline	42	2.126	2.774	37	2.573	3.331
	Endpoint (12 weeks)	39	2.438	3.634	35	2.014	2.586
ESR (mm/h)	Baseline	43	7.814	5.779	42	8.405	7.487
	Endpoint (12 weeks)	39	7.462	5.190	37	7.324	7.196
IL6 (pg/ml)	Baseline	43	1.765	0.710	40	1.696	0.565
	Endpoint (12 weeks)	39	1.806	0.760	36	1.602	0.484
IL1B (pg/ml)	Baseline	43	0.560	0.251	40	0.533	0.234
	Endpoint (12 weeks)	39	0.536	0.228	35	0.516	0.190
TNFa (pg/ml)	Baseline	43	1.615	0.396	41	1.600	0.464
	Endpoint (12 weeks)	39	1.513	0.240	37	1.525	0.296
**Oxidative Stress**							
8OHdG(ng/mL)	Baseline	43	5.181	1.533	41	4.923	1.641
	Endpoint (12 weeks)	39	5.022	1.251	37	4.891	1.337
teinCarbonyls (nmol/mg)	Baseline	43	4.769	1.614	41	5.180	1.788
	Endpoint (12 weeks)	39	4.675	1.537	36	5.165	1.725
**Neuronal Function**							
BDNF (pg/ml)	Baseline	43	24275.174	7721.800	41	23587.705	6435.074
	Endpoint (12 weeks)	39	23846.659	5250.689	36	22789.782	5630.611
AB40 (pg/ml)	Baseline	43	180.652	85.298	38	156.301	48.586
	Endpoint (12 weeks)	39	177.109	69.749	33	161.655	47.180
AB42 (pg/ml)	Baseline	13	90.295	44.638	11	166.106	157.325
	Endpoint (12 weeks)	12	86.528	31.517	10	171.518	139.650

Table S7. Medians and interquartile ranges

	Placebo			Curcumin		
	Median	25th	75th	Median	25th	75th
STAI-T	29	24	34	27	22	31.25
BDI	3	1	5	2	0	6

	Placebo			Curcumin		
	Median	25th	75th	Median	25th	75th
CHG_ANXIOUS_V1	5	-2	19	4	-2.5	18
CHG_ANXIOUS_V2	4	-2.5	9.5	1	-3	11.5
CHG_ANXIOUS_V3	4	-3	12	2	-3	13.25
CHG_CONCENTRATION_V1	-40	-59	-7	-48.5	-69	-31
CHG_CONCENTRATION_V2	-29	-61	-0.5	-41	-65.5	-14
CHG_CONCENTRATION_V3	-47	-63.5	-11.75	-45.5	-57.5	-17.5
CHG_FATIGUE_V1	9	0	22	14	2	29.5
CHG_FATIGUE_V2	2	-4	21.5	2	-5.5	11.5
CHG_FATIGUE_V3	5	-5.5	14	3	-5	13.25

	Placebo			Curcumin		
	Median	25th	75th	Median	25th	75th
GHQ_SomaticSymptoms_V1	3	2	7	3	2	7
GHQ_SomaticSymptoms_V2	3	2	6.5	4	2	5.5
GHQ_SomaticSymptoms_V3	4	2	7	3	2	7
GHQ_AnxietyInsomnia_V1	4	2	6	4	2	7
GHQ_AnxietyInsomnia_V2	4	3	7	3	1	5
GHQ_AnxietyInsomnia_V3	3	2	5	3	1	6
GHQ_SocialDysfunction_V1	7	6	8	7	6	7
GHQ_SocialDysfunction_V2	7	6	7	7	6	7
GHQ_SocialDysfunction_V3	7	6	7	7	6	7
GHQ_Depression_V1	0	0	1	0	0	1
GHQ_Depression_V2	0	0	0	0	0	0
GHQ_Depression_V3	0	0	0	0	0	0
GHQ_Total_V1	14	11	19	14.5	10	20.25
GHQ_Total_V2	13	11	19	14	9.5	18.5
GHQ_Total_V3	14	12	18	12	10	19
CFS14_Total_V1	14	13	15	14	14	16
CFS14_Total_V2	14	13	16	14	13	14.5
CFS14_Total_V3	14	14	18	14	14	15
CFS14_PhysicalFatigue_V1	8	7	8.25	8	8	9.25
CFS14_PhysicalFatigue_V2	8	7.5	9	8	8	8
CFS14_PhysicalFatigue_V3	8	8	10	8	8	8
CFS14_MentalFatigue_V1	6	5.75	6	6	6	6
CFS14_MentalFatigue_V2	6	6	6.5	6	5.5	6
CFS14_MentalFatigue_V3	6	6	7	6	6	6
POMS_TMD_V1	2	-11	17	3	-11.5	10.5
POMS_TMD_V2	3.5	-6.75	20.75	-1	-16.5	10.5

POMS_TMD_V3	6	-12	15	-2	-10	6
POMS_AngerHostility_V1	3	0	6	2	1	5
POMS_AngerHostility_V2	3	1	6.5	2	0	4
POMS_AngerHostility_V3	2	0	4	1	0	4
POMS_ConfusionBewilderment_V1	4	2	8	4	3	6
POMS_ConfusionBewilderment_V2	5	2	7	3	2	5
POMS_ConfusionBewilderment_V3	5	2	7	3	2	6
POMS_DepressionDejection_V1	1	0	5	2	0	3.5
POMS_DepressionDejection_V2	3	1	6.75	2	0	5
POMS_DepressionDejection_V3	2	0	3	2	0	3
POMS_FatigueInertia_V1	5	2	6	5.5	2	8
POMS_FatigueInertia_V2	5	3	10.5	4	0.5	7
POMS_FatigueInertia_V3	6	4	11	5	2	7
POMS_TensionAnxiety_V1	5	3	8	5	2	7
POMS_TensionAnxiety_V2	5	3	8	3	1.5	6
POMS_TensionAnxiety_V3	4	3	7	4	1	6
PSS_V1	10.5	8	14.25	11	8	15
PSS_V2	11	6.5	15	11	7.5	15
PSS_V3	10	7	14	11	8	14
PSQI_C1_Subjective_Quality_V1	1	1	1	1	0	1
PSQI_C1_Subjective_Quality_V2	1	0.25	1	1	0	1
PSQI_C1_Subjective_Quality_V3	1	0	1	1	1	1
PSQI_C2_SleepLatency_V1	1	0	1	1	0	1
PSQI_C2_SleepLatency_V2	1	0	1	0	0	1
PSQI_C2_SleepLatency_V3	1	0	1	1	0	1
PSQI_C3_Sleep_Duration_V1	0	0	1	0	0	0.5
PSQI_C3_Sleep_Duration_V2	0	0	1	0	0	0.5
PSQI_C3_Sleep_Duration_V3	0	0	1	0	0	1
PSQI_C4_Sleep_Efficacy_V1	1	0	3	0	0	2
PSQI_C4_Sleep_Efficacy_V2	1	0	3	1	0	1
PSQI_C4_Sleep_Efficacy_V3	1	0	2	1	0	2
PSQI_C5_Sleep_disturbances_V1	1	1	2	1	1	2
PSQI_C5_Sleep_disturbances_V2	1	1	2	1	1	2
PSQI_C5_Sleep_disturbances_V3	1	1	2	1	1	2
PSQI_C6_Use_of_Medication_V1	0	0	0	0	0	0
PSQI_C6_Use_of_Medication_V2	0	0	0	0	0	0
PSQI_C6_Use_of_Medication_V3	0	0	0	0	0	0
PSQI_C7_Day_Dysfunction_V1	1	1	1	1	1	1
PSQI_C7_Day_Dysfunction_V2	1	1	1	1	1	1
PSQI_C7_Day_Dysfunction_V3	1	1	2	1	1	1
PSQI_Global_Score_V1	5	3	7.5	5	3	7
PSQI_Global_Score_V2	5.5	4	7	4	3	7.5
PSQI_Global_Score_V3	5	4	7	6	3	7

	Placebo			Curcumin		
	Median	25th	75th	Median	25th	75th
DATT_TotalRT_V1	1529	1385	1671	1476.5	1395	1667.75
DATT_TotalRT_V2	1536	1371.5	1695.5	1453	1381.5	1615.5

DATT_TotalRT_V3	1525.5	1375	1675.25	1507	1375.5	1601.25
DATT_FocusedRT_V1	1486	1309	1635	1491	1367.75	1598.75
DATT_FocusedRT_V2	1504	1331	1652.5	1424	1345.5	1553.5
DATT_FocusedRT_V3	1472.5	1359.5	1588	1495.5	1380.25	1613.25
DATT_DividedRT_V1	1514	1390	1688	1521	1375.75	1766.25
DATT_DividedRT_V2	1544	1375.5	1678	1457.5	1386	1638.75
DATT_DividedRT_V3	1548.5	1410.25	1718.5	1536.5	1396.75	1643.25
DATT DivRT-FocRT V1	26	-47	98	53	5.75	156.5
DATT DivRT-FocRT V2	54	-58	160.5	42	-47	127
DATT DivRT-FocRT V3	70.5	-11.5	168.75	50	-27.25	128.25

	Placebo			Curcumin		
	Median	25th	75th	Median	25th	75th
AF_ACC_Con_vs_Neu_V1	0	0	0	0	-2.78	0
AF_ACC_Con_vs_Neu_V2	0	0	0	0	-2.78	2.78
AF_ACC_Con_vs_Neu_V3	0	-2.09	0	0	0	2.78
AF_Con_RT_V1	587	533	625	574.5	546.5	618.5
AF_Con_RT_V2	571	534.5	641	575	535	618.5
AF_Con_RT_V3	567	524	642	555.5	539.5	601.75
NASA_PhysicalDemand_V1	25	12	39	22.5	12	39.25
NASA_PhysicalDemand_V2	21	9.5	52.5	22	14	45
NASA_PhysicalDemand_V3	22	10.25	40.5	23.5	13.5	36.25

	Placebo			Curcumin		
	Median	25th	75th	Median	25th	75th
Urea_V1	5.80	5.00	6.80	5.60	4.58	6.93
Urea_V3	5.90	4.70	6.70	5.90	5.10	6.80
UricAcid_V1	0.34	0.28	0.37	0.31	0.24	0.37
UricAcid_V3	0.33	0.27	0.39	0.32	0.23	0.38
Calcium_V1	2.33	2.31	2.38	2.31	2.25	2.35
Calcium_V3	2.31	2.28	2.38	2.32	2.27	2.39
Adj._Ca._V1	2.35	2.33	2.41	2.33	2.28	2.40
Adj._Ca._V3	2.34	2.31	2.38	2.34	2.29	2.38
Albumin_V1	38.00	37.00	40.00	38.00	37.00	40.00
Albumin_V3	39.00	37.00	41.00	39.00	38.00	40.00
ALP_V1	70.00	61.00	82.00	66.00	56.75	83.00
ALP_V3	70.00	59.00	77.00	65.00	54.50	75.50
Bilirubin_V1	11.00	8.00	12.00	10.50	8.00	16.25
Bilirubin_V3	10.00	8.00	12.00	11.00	8.00	15.00
GGT_V1	19.00	14.00	27.50	20.00	13.00	27.75
GGT_V3	18.00	14.00	30.00	18.00	11.00	24.00
AST_V1	20.00	18.00	23.00	20.50	19.00	23.00
AST_V3	20.00	17.75	23.00	23.00	19.00	24.75
ALT_V1	17.00	14.00	24.50	16.00	14.00	20.50
ALT_V3	18.00	14.00	26.00	18.00	14.25	24.00
Glucose_V1	5.30	5.10	5.73	5.20	4.90	5.70
Glucose_V3	5.10	4.80	5.60	5.30	4.90	5.85

Triglyceride_V1	1.10	0.80	1.63	0.90	0.70	1.50
Triglyceride_V3	1.05	0.70	1.63	1.00	0.70	1.45
Total_Chol_V1	4.90	4.40	6.10	5.25	4.13	6.10
Total_Chol_V3	5.10	4.50	5.80	5.30	4.15	6.05
NonHDL_Chol_V1	3.60	3.00	4.40	3.30	2.58	4.10
NonHDL_Chol_V3	3.50	3.10	3.95	3.20	2.60	4.25
LDLHDL_Ratio_V1	2.00	1.60	2.70	1.70	1.30	2.00
LDLHDL_Ratio_V3	2.00	1.48	2.43	1.80	1.30	2.18
CholHDL_Ratio_V1	3.50	2.90	4.00	3.00	2.50	3.40
CholHDL_Ratio_V3	3.40	2.80	4.00	3.05	2.43	3.68
hsCRP_V1	0.75	0.38	2.98	0.70	0.30	3.50
hsCRP_V3	0.80	0.30	2.30	0.70	0.30	3.20
ESR_V1	7.00	5.00	11.00	5.00	5.00	9.75
ESR_V3	5.00	5.00	12.00	5.00	2.00	8.00
IL6_V1	1.58	1.22	2.22	1.53	1.34	1.90
IL6_V3	1.56	1.31	2.22	1.45	1.25	1.82
IL1B_V1	0.46	0.41	0.70	0.49	0.36	0.61
IL1B_V3	0.46	0.42	0.61	0.49	0.38	0.61
TNFa_V1	1.57	1.36	1.73	1.51	1.37	1.69
TNFa_V3	1.48	1.38	1.56	1.48	1.30	1.61
8OHdG_V1	5.06	3.85	6.21	4.52	3.77	5.86
8OHdG_V3	4.80	4.04	5.84	4.45	3.90	5.81
BDNF_V1	23792.90	19040.91	28389.71	21520.88	18912.77	28580.81
BDNF_V3	22506.03	20368.97	27036.68	21745.27	18642.58	27349.64
AB40_V1	155.18	128.63	191.39	145.77	119.15	177.66
AB40_V3	153.03	141.68	198.84	157.61	128.46	185.14
AB42 V1	79.08	62.40	93.46	87.47	76.28	267.84
AB42 V3	75.58	61.74	108.05	91.59	78.72	266.54
